# Promoting expression in gene therapy: more is not always better

**DOI:** 10.1038/s44321-024-00036-y

**Published:** 2024-02-27

**Authors:** Maria M Zwartkruis, Ewout JN Groen

**Affiliations:** 1 Department of Neurology, UMC Utrecht Brain Center, University Medical Center Utrecht, Heidelberglaan 100, 3584 CX Utrecht, The Netherlands; 2 Department of Genetics, University Medical Center Utrecht, Heidelberglaan 100, 3584 CX Utrecht, The Netherlands

**Keywords:** Genetics, Gene Therapy & Genetic Disease, Musculoskeletal System

## Abstract

E. Groen and M. Zwartkruis discuss improved gene therapy for spinal muscular atrophy as reported by J. Xie and colleagues, in this issue of *EMBO Mol Med*.

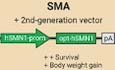

## Teaser

Spinal muscular atrophy (SMA) is an autosomal recessive neuromuscular disease caused by loss-of-function of *SMN1*. SMA is characterized by degeneration of motor neurons in the spinal cord, leading to progressive muscle weakness and atrophy. One of three currently available treatments is onasemnogene abeparvovec, an AAV9-based gene replacement therapy. Despite its effectiveness in improving motor function in SMA patients, its long-term safety profile remains unclear and adverse events such as liver toxicity are common. This may be caused by high vector dose or supraphysiological levels of SMN, driven by its strong, ubiquitous promoter. In this issue of *EMBO Molecular Medicine*, Xie et al addressed this by replacing the promoter of a benchmark virus—equivalent to onasemnogene abeparvovec—by an endogenous *SMN1* promoter. In a common mouse model of SMA, treatment with this 2nd generation vector restored SMN expression close to physiological levels across tissues, resulting in improved safety and efficacy. This approach holds promise for safer and more efficacious AAV gene therapy for SMA and other diseases.

SMA is caused by biallelic mutation of *SMN1*, coding for the ubiquitous SMN protein. For SMN production, SMA patients instead rely solely on its paralog *SMN2*, which produces variable but insufficient amounts of full-length mRNA. Currently, three therapies are available for SMA: two of these—nusinersen and risdiplam—increase SMN protein expression by correcting splicing of *SMN2* transcripts. A third therapy, onasemnogene abeparvovec, is a single-dose systemic AAV9-based gene therapy replacing the missing *SMN1* gene. It is currently approved for patients with genetically confirmed SMA who are younger than 2 years (Mercuri et al, 2022) and has now been given to over 3000 patients according to its manufacturer.

When administered early—ideally presymptomatically—onasemnogene abeparvovec is highly effective in improving survival and motor function of SMA patients. However, we still have a limited understanding of its long-term safety profile. In preclinical studies, long-term AAV9-mediated SMN overexpression may trigger SMA-like pathology in mice (Van Alstyne et al, 2021) and cause liver damage and sensory deficits in large animal models (Hinderer et al, 2018), although variation in vector type and transgene composition may complicate direct comparisons and translatability of these observations. In patients, liver-associated adverse events were reported in 34% of 100 patients from five clinical trials (Chand et al, 2021). Possible explanations for these adverse events could be either an immune response to the high dose of AAV9, or supraphysiological *SMN1* levels driven by the strong cytomegalovirus enhancer/chicken β-actin (*CMVen/CB*) promoter. As SMN protein expression differs per tissue in both humans and mice, and is normally tightly regulated (Ramos et al, 2019; Groen et al 2018), it is challenging to control transgene expression so that it is similar to physiological levels. Indeed, onasemnogene abeparvovec treatment leads to widespread, high expression of SMN in human neuronal and peripheral tissues (Thomsen et al, 2021).

In this issue of *EMBO Molecular Medicine*, Xie et al aimed to reduce toxicity of AAV9-mediated gene therapy in SMNΔ7 mice, a common SMA mouse model. First, they attempted this by reducing the amount of vector while maintaining efficacy. To do this, they replaced the *SMN1* coding sequence (ori-*hSMN1*) from their benchmark vector – similar to onasemnogene abeparvovec – with a codon-optimized human *SMN1* (opt-*hSMN1*) sequence, resulting in 3-fold increased SMN protein levels compared to the benchmark construct. Surprisingly, treatment of SMA mice with similar or lower doses of this vector led to reduced survival compared to the benchmark vector. Hepatotoxicity was observed in both SMA mice and healthy littermates that received the codon-optimized vector. Suppressing SMN transgene expression in the liver specifically by adding miR-122 binding sites to the vector, led to reduced SMN expression and hepatotoxicity, suggesting that toxicity associated with supraphysiological SMN levels may compromise therapeutic results (Xie et al, 2024).

**Figure 1:**
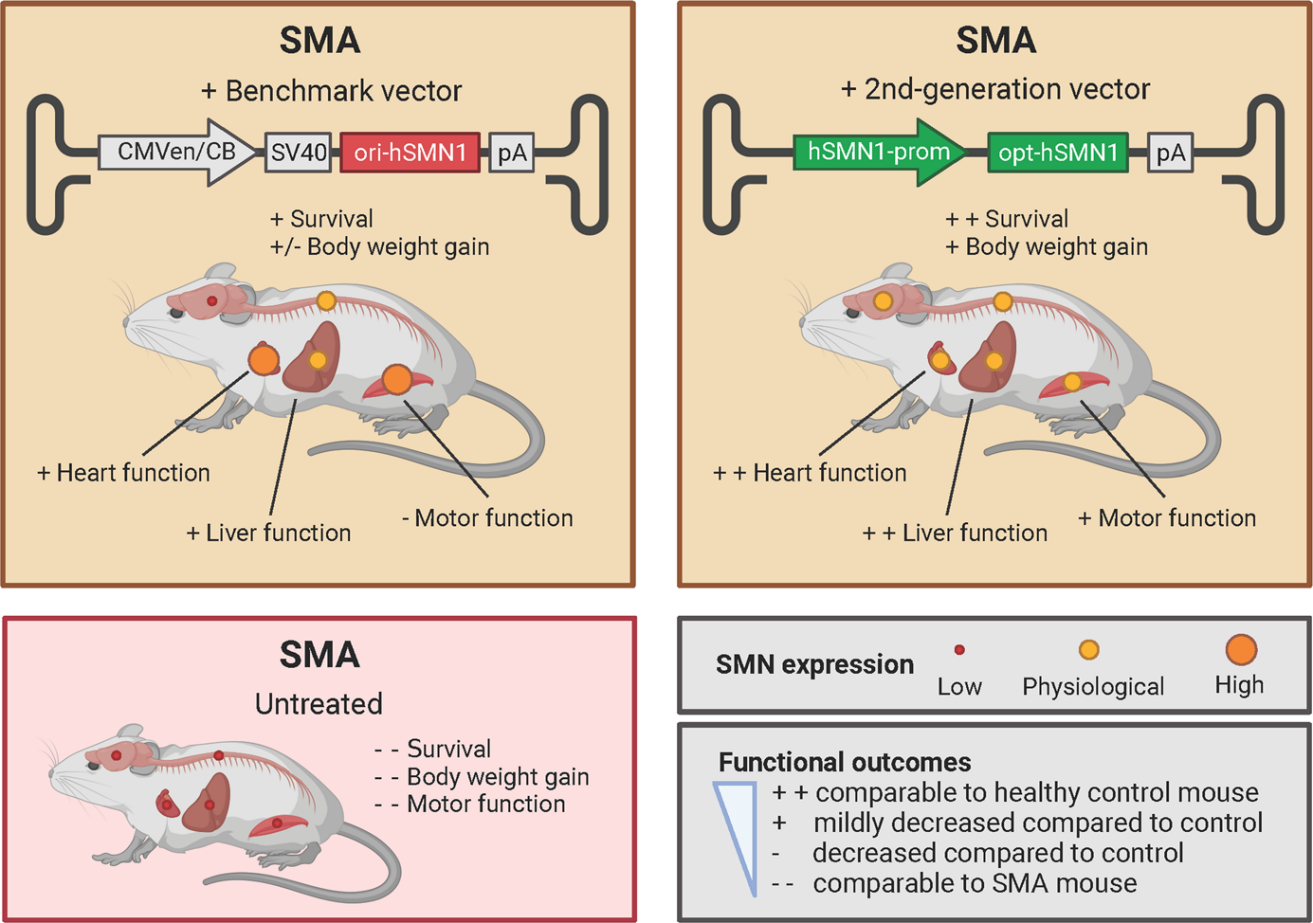
The 2nd generation vector rescues SMN expression to a physiological level in different organs. It rescues SMA symptoms more effectively and shows fewer signs of toxicity than the benchmark vector. SMN expression is shown schematically for different organs; at P30 for liver and P90 for other organs. For comparative purposes, SMN expression in SMA mice (Taiwanese and 2B/- mouse model at P8) is also shown (Groen et al, 2018), although it was not studied in this article. pA: polyadenylation sequence; SV40: SV40 intron. Created with BioRender.com.

To achieve a more physiological expression pattern of SMN, Xie et al decided to next replace the promoter of their codon-optimized vector with a derivative of the endogenous promoter of *SMN1* (*hSMN1*-prom, Fig. 1). Treatment of SMA mice at P0 with this 2nd generation vector resulted in better survival rates and higher weight gain than the benchmark vector. Treatment at P5 had the same effect to a lesser extent, indicating that the therapeutic window is bigger, but that treatment at an earlier time point is still more effective. Animals treated with the 2nd generation vector also showed improved motor function and reduced neuromuscular pathology. A possible explanation for this may be that SMN expression patterns in brain and spinal cord and across cell types are more like healthy carriers after treatment with the 2nd generation vector (Xie et al, 2024).

Peripherally, compared to control, both heart and liver function remained lower in SMA mice after treatment with the benchmark vector, but improved to levels similar to control after treatment with the 2nd generation vector. A possible explanation for this is SMN expression level in peripheral organs: 90 days after treatment, SMN levels across organs were similar to those of healthy carriers in 2nd generation-treated mice but remained much higher in benchmark-treated mice. Finally, fewer 2nd generation-treated mice showed ear necrosis, watery eyes or diarrhea than benchmark-treated animals. Altogether, these results point towards an increased safety and efficacy of gene therapy when using an endogenous promoter, rather than a constitutive promoter (Xie et al, 2024).

With its high effectiveness, onasemnogene abeparvovec serves as a great example for development of gene therapies for other neurological diseases (Ling et al, 2023). Often, the focus of gene therapy development has been to maximally increase gene expression. However, the long-term impact of ubiquitous and constitutive overexpression proteins after gene therapy remains uncertain, posing possible safety concerns. The current study provides a significant step forward on the path towards a new generation of gene therapies that could eliminate these concerns. However, AAV9 expression in mouse models is not always directly translatable to humans. This 2nd generation vector will therefore also need further testing in more models of SMA (Signoria et al, 2023), including non-human primates, and eventually in clinical trials.

This study also highlights that, despite an urgent medical need in some cases, it may be beneficial to explore different optimization options for gene therapies before bringing them to the market. Once approved therapies are available—as for SMA—finding participants for clinical trials may become challenging: for patients or parents, it may be difficult to choose experimental treatments over approved treatments with proven effectiveness. However, developments as those described by Xie et al provide a promising blueprint of gene therapy development for diseases for which no effective treatments are available.

In conclusion, Xie et al provide an important insight on the importance of physiological transgene expression in gene therapy, and a proof-of-concept that this can be reached with endogenous gene promoters.
